# A focused netnographic study exploring experiences associated with counterfeit and contaminated anabolic-androgenic steroids

**DOI:** 10.1186/s12954-020-00387-y

**Published:** 2020-06-12

**Authors:** Evelyn Frude, Fiona H. McKay, Matthew Dunn

**Affiliations:** 1 School of Health and Social Development, Deakin University, Geelong Waterfront Campus, Locked Bag 20000, Geelong, Victoria 3220 Australia; 2 National Drug and Alcohol Research Centre, University of New South Wales, Sydney, New South Wales Australia

**Keywords:** Steroids, Counterfeit, Fake, Contaminated, Online forums, Anabolic-androgenic steroids, Internet forum

## Abstract

**Background:**

A primary consequence of illicit drug markets and the absence of regulation is the variable quality or purity of the final product. Analysis of anabolic-androgenic steroid seizures shows that these products can contain adulterated products, product not included on the label, or product of unsatisfactory standard. While the potential negative effects of counterfeit anabolic-androgenic steroids (AAS) use is a recognised risk associated with use, no study has explored personal experiences associated with use. The aim of the present study was to use online discussion forums to investigate and explore the experiences associated with the purchase and consumption of counterfeit AAS among consumers.

**Methods:**

An online search was conducted to identify online forums that discussed counterfeit or contaminated AAS; three were deemed suitable for the study. The primary source of data for this study was the ‘threads’ from these online forums, identified using search terms including ‘counterfeit’, ‘tampered’, and ‘fake’. Threads were thematically analysed for overall content, leading to the identification of themes.

**Results:**

Data from 134 threads (2743 posts from 875 unique avatars) was included. Two main themes were identified from the analysis: (1) experiences with counterfeit product and (2) harms and benefits associated with counterfeit product.

**Conclusions:**

The use of counterfeit or contaminated substances represents a public health concern. Those who report using performance and image enhancing drugs such as AAS for non-medical purposes report consuming these substances and experiencing harm as a result. Consumers take steps to limit coming into contact with counterfeit or contaminated product, though recognise that many of these have limitations. The implementation of accessible drug safety checking services may provide an opportunity to provide consumers with information to assist them with making healthier choices.

## Background

A primary consequence of illicit drug markets and the absence of regulation is the variable quality or purity of the final product [1], and for those substances which are illegal or used non-medically, there is a risk that substances may be of unknown quality or contain unknown impurities [2]. Counterfeit drugs have become a global problem and have increased with the expansion of the Internet [3–5]. Counterfeit drugs are considered by the Australian Therapeutic Goods Administration (TGA) and the World Health Organization (WHO) as those which are substandard and falsified, and either fail to meet quality standards or are deliberately manufactured to imitate a legitimate product [6, 7]. They may contain no active ingredient, the wrong active ingredient, or an incorrect amount of the correct active ingredient. These products can have serious health consequences including poisoning and kidney and liver damage [8]. The WHO [7] has declared counterfeit drugs an unacceptable risk to public health, with consumers in low-income countries thought to be at a 10% risk of experiencing counterfeit products, with many users unable to afford lifesaving drugs from medical practitioners [9]. In high-income countries, up to 1% of consumers are negatively affected by counterfeit products [7]. Consumption of counterfeit drugs can result in the drugs being ineffective to treat medical conditions which can deteriorate and may result in death [3, 8].

The use of counterfeit products is a risk for those who engage in the non-medical use of anabolic-androgenic steroids (AAS). AAS are synthetic forms of the hormone testosterone and are scheduled or controlled medications in many countries where they are used under medical supervision to treat a number of health conditions, primarily those related to testosterone deficiencies [10]. They are also used non-medically by elite and non-elite athletes for a number of positive performance and body image effects, including increased muscle mass, increased strength, decreased body fat, decreased recovery time, improved athletic ability, and a more defined physical appearance [11]. Studies analysing seizures of AAS and other performance and image enhancing drugs (PIEDs) have found that a sizeable proportion of seizures are adulterated [12], do not contain the substance stated on the label [13–15], contain substances not declared on the label [16], or are of pharmaceutical quality of an unsatisfactory standard [15, 17–19]. The use of such product poses a health risk to consumers, who have reported harms such as infections at injecting sites when counterfeit or contaminated products are used [20].

The use of contaminated and counterfeit products appears to be an anticipated risk, with consumers reporting the adoption of a number of strategies to address this concern. These include checking packaging to ensure the product has not been tampered with, or purchasing through trusted sources [20]; poor-quality product in the bodybuilding community has been cited as a reason why some have commenced supplying PIEDs to other consumers [21]. Some consumers report importing the raw materials and manufacturing their own product [3], a process known as ‘homebrewing’ [22]. This suggests that the harm related to counterfeit product is an anticipated risk related to AAS use, and consumers take steps to alleviate or mitigate against this risk.

To explore the experiences of this known risk and potential risk mitigation strategies, this study employs a netnographic approach to explore online discussions between PIED consumers regarding their concerns relating to the use of contaminated or counterfeit AAS. The importance of online forums for discussing and disseminating information related to PIEDs such as AAS has been discussed in detail elsewhere [23]. Online forums provide those who engage in illicit or stigmatised behaviours an anonymous environment to exchange information and experiences, while fostering a sense of community [24, 25]. This environment can allow people to seek information from a large community without having to identify themselves, or enter into an environment that may pose risks [26, 27]. These forums have become an online extension of the real-world interactions that have been important in PIED consuming communities [23].

This study is underpinned by the work of Lupton and Tulloch [28] who suggest that some individuals participate in voluntary risk-taking, participating in activities that are perceived to be at least somewhat dangerous. They suggest that individuals who engage in voluntary risk-taking view concepts of danger, uncertainty, threat, and hazard in a positive, rather than negative, way. Risk-takers have a sense of achievement when they complete an activity without experiencing a negative consequence [28]. Lupton and Tulloch [28] conclude that there are three dominant reasons people choose to participate in risky behaviour, namely self-improvement, emotional engagement, and control. By applying this theory to the non-medical use of AAS, we suggest that AAS consumers receive positive reinforcement for their risk-taking behaviours when they achieve the goals underpinning their use. Further, use of AAS and the effects they can have on an individual’s body may lead to the perception that the consumer has a greater sense of control over their body.

Research has investigated risk-taking behaviours of AAS users. This research suggests that use of AAS is a predictor of other health-risk behaviours [29]. Much of this research has been focused on the use of AAS by teenagers or adolescents. For example, Lorang and colleagues [30] suggest that young steroid users are ‘thrill seekers’ who ‘do not recognize the potential risks of their actions’ (p 367), while Ravn and Coffey [31] describe AAS use as a ‘lifestyle’ or ‘identity’ risk or choice, and therefore not considered as serious as other drug-taking risks. This is consistent with other work from Lupton [32] which states that there is a link between emotions and the decision-making process when considering risk, with the suggestion that feelings and risk analysis occur simultaneously [32]. However, if death or serious illness or injury is not perceived to be a real consequence with significant risk (i.e. something that it is likely to occur), AAS consumers may not take this into consideration. Applying this theory, we posit that AAS users will continue to source and use the drug illicitly despite knowing its associated risks. While the potential negative effects of counterfeit AAS use is a recognised risk associated with use, no study has explored personal experiences associated with use. The aim of the present study was to use online discussion forums to investigate and explore the experiences associated with the purchase and consumption of counterfeit AAS among consumers.

## Methods

A netnographic approach was used as a guide to explore the experiences associated with the purchase and consumption of counterfeit AAS and PIED products among consumers. Netnography involves using the naturally occurring conversations that are recorded on publicly available digital networks, such as online discussion forums, as data that can be used to explore a specific cultural group and investigate consumer behaviour [33, 34]. Researchers are able to observe how fellow consumers influence and inform each other about available products and the potential consequences of use through these forums [34]. Netnography has been used as a research method in research dealing with sensitive health and medical issues in which participants may feel uncomfortable disclosing their personal information to a research team [35, 36]. These include those in the alcohol and other drugs sector and more specifically those associated with AAS use [23, 37]. The approach used in this project has a specific focus, seeking to explore the experiences associated with counterfeit AAS and PIED products among a population of consumers who frequent online discussion forums.

### Data collection

The study followed the method employed by Tighe et al. [23] in their study of PIED consumers’ use of online forums. Google.com.au was used to identify Australian-based online forums that discussed PIED use, with sites chosen based on their Google search ranking. Search terms included ‘Australian bodybuilding forums’, ‘steroid chat rooms Australia’, and ‘performance-enhancing drugs Australia’. To be included in the study, sites were required to be moderated, have a forum that was regularly used (i.e. daily posts by users), be publicly accessible and searchable, and be open to PIED use (demonstrating some sort of advice, education, and/or acknowledgement to health in using PIEDs). Sites were excluded if they were not Australian-based, were largely news based, or predominantly served as product advertisement. Based on the inclusion and exclusion criteria, three online forums were included in this study.

The primary source of data for this study is the ‘threads’ from the online forums. Threads consist of strings of posts that are connected by a central theme. Threads can be considered to be discussions, with the structure of an online forum allowing for a community’s discussion history to be archived and searched and later retrieved [38]. Depending on the structure of the forum, all threads from the main board or all threads from related sub-boards (e.g. Anabolic Steroids, Steroids, and Performance Enhancers) were searched. Following the method used by Hutchinson et al. [38] and Tighe et al. [23], the first five pages or first 20 threads (whichever was applicable, depending on how the forum site was designed) of the forum or sub-forum were read followed by (sub) threads. If the title directly or indirectly suggested that the thread would contain discussions of counterfeit/contaminated AAS, it was inspected. If this was confirmed, the full thread was captured for analysis. Additional threads were identified using the search terms ‘counterfeit’, ‘fake’, and ‘tampered’ in the site’s search function. Participation in the discussions on the website requires the creation of an individual avatar, which are used for identification between members. Each avatar has a ‘handle’, or nickname, and an individual may choose to reveal as much or as little about themselves as they wish, including a picture of themselves, their geographical location, age, and gender. Only handles were downloaded, though these have been excluded from quotes.

Forums were monitored from February 2019 onwards, with data collection occurring from June to July 2019. Temporal restrictions were applied; threads prior to 2010 were not considered for inclusion as evidence suggests that in 2010 the use of PIEDs, especially among new consumers, increased [39]. The data were collected in a covert manner, meaning that data collection was unobtrusive to the users of the sites and only included data that was pre-existing; no additional data was sought [33]. Previous research has promoted this form of non-participant observation with concerns that researcher engagement may negatively influence the nature of the conversation [40, 41]. As these forums are publicly available, the creation of an account and avatar was not required to access the threads.

### Data analysis

Data were analysed following the five-step Empirical Phenomenological Psychological (EPP) approach developed by Karlsson [42]. The EPP method has previously been used in a variety of netnographic studies including those that involve the subject area of alcohol and other drugs [43, 44] and more specifically AAS use [23]. Once the relevant threads were identified, they were transferred to a word document for analysis following the EPP protocol. After the documents had been created, they were uploaded to the qualitative data analysis computer software package NVivo version 12 to be analysed [45].

The five-step analysis process proceeded as follows: In step 1, the first author read the data three times to become familiar with the data with the aim of minimising researcher bias towards the objectives. In step 2, the first author began coding the data whenever a new focus or topic was identified. This did not take syntax into consideration. In step 3, the first author then collaborated with the other authors to collectively determine the true meanings of posts. This process is known as researcher triangulation, a procedure employed to enhance research rigour [46]. In step 4, sub-themes were then confirmed by the research team with any necessary changes due to disagreement being made. The final step (step 5) involved the coded data (sub-themes) being categorised into broader themes. Relevant themes were those that related to the objectives and main aim of the study as well as Lupton and Tulloch’s [28] risk theory and its relation to the topic, specifically surrounding the purchase and consumption of counterfeit AAS or PIED, and some discussion of harms, experienced as a result of using these products.

Threads were analysed following the constant comparative method advocated by Miles and Huberman [47]. Threads were read and re-read for content familiarity and to allow for the identification of these overarching themes. Themes were reviewed across forums and checked for distinction and coherence. An ethics exemption was granted by Deakin University Human Research Ethics Committee.

## Results

In total, 134 threads (40 from site 1, 38 from site 2, and 56 from site 3) and 2743 individual posts from 875 unique avatars were included in the analysis. Threads as a whole were analysed to identify the types of information forum members sought and shared. Two overarching themes were identified from this analysis and are presented below and supported by quotes, which are presented verbatim and unedited.

### Theme 1: Experiences with counterfeit product

The first theme is concerned with discussions that focus on personal experiences with counterfeit product. Within this theme, forum members discussed the products they had used which they believed were counterfeit and how they determined whether a product was counterfeit.

Forum members reported that they had purchased, received, and consumed counterfeit AAS or other PIED products including tablets, capsules, injectable products, oils, or gels. Discussions often commenced as cautionary, with members warning others about counterfeit products in circulation, before becoming focused on the potential harms that could result from using that substance.

Member 255—‘Becareful with the varcap25 as there is 100s of containers getting around claiming to be 25mg and there actually 10mg ones.. Would love to get my hands on some meditech ones here there awesome’

Member 725—‘I mean, you don't even know how much to dose and Clen can be easily overdosed esp the gel.’

A counterfeit product was considered one that contained less, more, or none of the active ingredient that was desired. Forum members claimed that they had received underdosed AAS or PIED products that had been watered down, thus containing less of the active ingredient than expected. There were also reports of AAS or PIED products being overdosed, determined by the subjective experience that the drug’s effect was stronger than expected. Some forum members also reported receiving products that contained a different active ingredient than what they had ordered. In these discussions, forum members would state their claims of counterfeit products but also reported a variety of methods used to decide whether the product was counterfeit. For some, a product was determined to be counterfeit because they had experienced an effect different to what they had expected, or experienced a side effect not related to the particular substance. For example, one forum member reported ordering the steroid stanozolol (brand name Winstrol®, used to promote muscle growth) but instead receiving methandrostenolone (brand name Dianabol®). The forum member had decided that they had received the wrong product by experiencing the side effect of gynecomastia, the swelling of the breast tissue.

Member 518—‘Fake winny [Winstrol]. winny can’t cause gyno.’

Others reported having their product tested after not experiencing the effects or side effects which they were expecting, with these tests revealing that the product contained a number of filler products to make up for the lack of active ingredient:

Member 131—‘Hey man I had the exact same ones had them lab tested this Is the honest truth they contained 2.5 mg of methylated testosterone and 2.5 mg of dianabol and they where supposed to be 10 mg tablets the rest was starch and filler there shit mate stay away unless u wanna double drop just to get 10 mg x4’

Other methods used to determine whether a product was counterfeit included relying on user experiences of particular products that were reported on the forums. This ranged from simple comments elicited from members about expectations to more advanced advice on the appearance of product. This included the actual product, such as the tablet, oil, or vial, as well as the packaging it came in, including the labelling and whether the seal appeared to have been tampered with. Forum members used this information provided by other trusted and experienced members as a guide to determine that a received product was counterfeit. The information provided helped to influence the decisions members made about consuming the product.

Member 203—‘I say this as a complete guess but i'd almost put my money on that being relabelled A.I, neither vial filled to the top, that same sort of cap that A.I have on their vials.’

Member 111—‘Looks good to me, try scratching the expiry off. If it comes off it is gtg.’

Informal testing methods can also help to identify if a product is counterfeit. For instance, forum members discussed placing the product in their mouth and tasting a different ingredient such as cornflour, or observing a liquid quickly dissolve, suggesting that a product was not legitimate. Formal methods were also suggested as ways to provide consumers with reassurance about whether a product was counterfeit. These included checking serial codes and completing tests on received products. Consumers reported using websites to check the serial codes of certain AAS and PIED products to ascertain legitimacy. One way in which leading pharmaceutical companies tried to thwart counterfeit manufactures replicating their product was to regularly update their product packaging. However, even these websites where the codes can be checked do not completely confirm the legitimacy of products. Counterfeit manufactures can replicate the packaging and labelling of products including copying the provided code, and products can be tampered with after they have been made in the distribution process.

### Theme 2: Harms and benefits associated with counterfeit product

The second theme was concerned with discussions that focused on the harms associated with consuming counterfeit products, but also what some forum members perceived as benefits stemming from use.

Discussions concerning harms related to use included those related to both physical health as well as mental health. Physical health effects included those which were almost considered nuisance effects, such as bloating, that was surmised to be caused by adulterants contained in the product. Effects that were more common included pain, abscesses, and infections related to injecting counterfeit product that some members believed was a result of the product being manufactured in unsterile environments.

Member 269—‘I know that the 6/15 was known to be a bad batch, but mine hurts my stomach (on empty) and gives me a really wierd feeling, Maybe its too strong for me.’

Forum members believed that products which were overdosed or contained a mix of overdosed and underdosed ingredients placed consumers at risk of experiencing heart issues or more serious consequences. Some members reported increasing their doses or a product that was believed to be underdosed; however, this was generally met with concern from others, with advice provided to try and purchase different products if there were doubts about strength or contamination:

Member 199—‘Gtg mate. But as (other forum member) said the darker ones are a lot more potent. The light ones still work (i've run a 14 week cycle on them in the past) but i just increased my intake by an extra tablet (extra 10 mg per day) to compensate.’

Member 752—‘Can't put a price on your health man Pretty expensive though, I'd just buy some chinese stuff and take 4ui’

In addition to these potential physical health consequences, mental health harms were discussed; however, this tended to focus not on harms resulting from counterfeit use, but from the anxiety that could result from *potentially* using counterfeit product. When trying to ascertain whether a product was ‘safe’, consumers are reporting ongoing anxiety and paranoia, and used the forums to try to alleviate these:

Member 218—‘Sweet cheers man, i just thought that being from ugl oz it would be a darker brown as all the tren I've seen from them has been quite dark, I know my source is legit as I've had heaps from him so I think I'm just being a bit paranoid.’

Another effect, considered a harm by forum members, was the frustration at spending large and unnecessary amounts of money on counterfeit AAS and PIED products that were ineffective and failed to provide them with the results they desired.

Member 255—I work in the mines in [jurisdiction] and let me just say there are some pissed off guys working out there paying top $$ for these and there underdosed to the max!! Im from [jurisdiction] and the guys that were on them have had very avg results even using 6 a day. Well I can tell u the labels have not been tampered with and the containers were 100% properly sealed so this is coming from the main players and by the looks of it there is 100,s if not 1000,s of these getting aound. Im only making people aware of this as when u spend the $$ u expect results. I do notice a lot of guys trying to push this product on here only to benefit themselves with some $$$ nice one guys..’

While counterfeit products were overwhelmingly associated with negative effects, a minority of forum members reported benefits related to their use. One of these benefits included the lower cost associated with counterfeit product relative to a ‘normal’ product which had been obtained through diverted avenues. A second benefit was effectiveness. Some forum members reported experiencing a better-than-expected effect from a product they consumed and thus assumed it had been overdosed, itself suggesting that it was a counterfeit product.

Member 250—‘As for AI tren,that is some of the most potent tren around. Might actually be overdosed because ive had swelling and inj site soreness after pinning it too. Corks at times but works fkn wonders...’

Member 265—‘Lots of counterfeits out there tho, Ive used counterfeit centrino and it was still really fucking good oil’

## Discussion

There are a number of issues associated with the non-medical use of PIEDs such as AAS, one of which has been identified as the risk of purchasing and consuming counterfeit products. Despite these risks, this is an under-researched area, with much of the literature focusing on the analysis of counterfeit product; little is known about the experiences of the consumers of these products. The aim of this study was to use online discussion forums to investigate and explore the experiences associated with the purchase and consumption of counterfeit and contaminated PIEDs. Forums members reported using a range of counterfeit and contaminated products, which were identified as such because they were perceived to contain less, more, or none of the active ingredient than expected, or lead to physical or mental health harms. Counterfeit and contaminated products were expected, and forum members took steps to identify them or to address any negative experiences. These findings provide insight into how PIED consumers respond to the potential harms which come from using substances for non-medical purposes and can inform harm reduction initiatives targeted towards this group.

The consumer experiences of coming into contact with contaminated or counterfeit product suggest that this is not an unusual or unanticipated aspect of PIED use and is line with studies that show a number of black-market seizures are under- or overdosed, contain no active ingredient, or contain ingredients different to those listed on the package [48], and are available in a number of forms including tablets and injectable products. Counterfeit products available in a variety of different forms across different drug categories have been reported in previous literature including tablets of alprazolam, antimalarial and anti-infective products, heroin, cosmetic injectable products, and peptide biopharmaceuticals [49–52]. There have also been reports of other drugs being replaced with different substances including heroin with fentanyl, and alprazolam with naxolone which have resulted in unintentional overdose [53, 54]. There is, however, a lack of research which has explored the short- and long-term effects experienced from using PIEDs which have been confirmed to contain adulterants, and this should be the focus of future research.

Forum members reported using a variety of methods to identify counterfeit products. Forum members were able to informally identify products they believed were counterfeit, and shared this information and advice on the forums, which included sharing details about what forum members believe products should look like including the packaging and labelling of the box as well as the appearance of the actual product. In a study conducted by Mhando and colleagues [55], which sought to increase public awareness and ability to identify antimalarial drugs, those who had a better knowledge on the health effects of counterfeit products were better able to distinguish between genuine and counterfeit antimalarial drugs through the inspection of the appearance of the drug and the accompanying packaging and labelling. This appears to be very similar in the current study, where the forum members acknowledged that counterfeit products were an issue of concern and that those who appeared to have more experience and knowledge about the issue were able to not only make informed decisions around identifying and consuming these products, but were able to share this with others.

While forum members did take steps to identify counterfeit or contaminated products, they rarely if at all mentioned using any type of presumptive testing kit. While these types of tests have their limitations, they may be able to provide an additional layer of information beyond the other steps forum members took, and which they admitted had limitations. There is a good body of evidence which shows that consumers of illicit substances will seek information regarding the content and purity of the substances they intend to take, and will modify their behaviour accordingly. Research investigating the use of drug safety testing (also known as drug checking, or occasionally pill testing) shows that consumers of MDMA (methylenedioxymethamphetamine) at music festivals and other events not only access these services, but modify their behaviours when informed that the purity of the substance they intend to consume may cause harm [56–58]. Making testing facilities easily accessible to PIED consumers could be one way to address harms associated with counterfeit and contaminated product. Furthermore, as drug safety testing facilities are staffed by harm reduction professionals, it may be an additional way to provide harm reduction and health advice to PIED consumers who may not normally be in a position to receive this information.

The Internet plays a pivotal role in enabling PIED consumers to access information, and online forums play an important part in knowledge dissemination among this group [23]. The discussions that took place on the online forums demonstrated that forum members were being proactive about their health. While the forums were Australian-based, it was clear that some members lived overseas and that even those who lived in Australia were using products purchased from foreign countries. The forums allowed members from different geographical locations who would not normally encounter each other in person to share advice and tips, a benefit listed in previous studies exploring forum use among hidden populations or those experiencing issues which can be surrounded by stigma [59, 60]. Members relied on others’ personal experiences, especially holding more senior members including moderators and administrators in high esteem as well as valuing consistency among multiple members’ opinions about certain products or brands. The more individuals who shared similar experiences and opinions, the more it appeared that members took on that advice when making decisions. This study also confirms the importance that this community places on social interactions, whether they be relationships formed offline or in the virtual space. Trust and acceptance as a member of the fitness or bodybuilding communities is an important step in acquiring knowledge from others [21, 61], and the distribution of PIEDs follows along similar lines, with social networks playing an important role in PIED supply and distribution [62]. Just as social networks play an important part in the distribution of PIEDs, so too does it play an important part in knowledge exchange.

This information sharing can be good and a way of helping to protect individuals from harm. In this sense, and in line with our previous research [23], forum members act as peers, providing harm reduction information to others in their community. Peers and their lived experiences are highly regarded in their communities and have been an important aspect of the harm reduction movement [63]; indeed, peers have been identified as a common source of information and help among those who use PIEDs such as AAS [64]. Online forums may provide a discreet, anonymous, and more readily accessible point of entry to peers and harm reduction information, and the lived experiences which can underpin this. The remaining challenges include how research—such as data on the purity and content of PIED seizures—can be introduced to these forums in a manner that makes these findings acceptable to the community, as well as how health professionals can use information from these forums and incorporate it into their work with consumers in real life.

### Limitations

A limitation of the study is that the sample was confined to discussions on three Australian discussion forums. The addition of more data from different sites, including international sites, may produce different findings. The data collected came only from forums which were in the public domain, and may be different from sites which have forums that have restricted access (e.g. on Facebook). This form of data collection also relies on the self-reported information and the personal experiences and opinions of the forum members, and removes the ability for researchers to ask follow-up or clarifying questions about the information which was posted. Researchers have conducted studies which have combined both virtual and offline discussions with consumers to combat the limitation of exploring virtual discussions alone [61, 65], and allow for researchers to explore the behaviour under question in greater depth. However, the importance placed on knowledge and the sharing of correct information within this community acts as a protective factor against any bias or incorrect information.

## Conclusions

The use of counterfeit or contaminated substances represents a public health concern. Through the analysis of discussion posts on Internet forums, this study found that those who use PIEDs such as AAS for non-medical purposes report consuming these substances and experiencing harm as a result. In line with previous research, consumers take steps to limit coming into contact with counterfeit or contaminated product, though recognise that many of these have limitations. The use of these forums provided forum members with the opportunity to receive harm reduction from peers. The implementation of accessible drug safety checking services may provide an opportunity to provide consumers with information to assist them with making healthier choices.

## Data Availability

The data analysed during the current study are available from the corresponding author on reasonable request.

## Abbreviations

AAS: Anabolic-androgenic steroids
MDMA: Methylenedioxymethamphetamine
PIEDs: Performance and image enhancing drugs
TGA: Therapeutic Goods Association
WHO: World Health Organization
